# A novel technique for the assessment of mechanical properties of vascular tissue

**DOI:** 10.1007/s10237-020-01292-w

**Published:** 2020-01-24

**Authors:** Stefan N. Sanders, Richard G. P. Lopata, Lambert C. A. van Breemen, Frans N. van de Vosse, Marcel C. M. Rutten

**Affiliations:** Department of Biomedical Engineering, Eindhoven University of Technology, PO Box 513, 5600MB Eindhoven, The Netherlands

**Keywords:** Ring-inflation, Constitutive modelling, Mechanical properties, Vascular tissue

## Abstract

Accurate estimation of mechanical properties of the different atherosclerotic plaque constituents is important in assessing plaque rupture risk. The aim of this study was to develop an experimental set-up to assess material properties of vascular tissue, while applying physiological loading and being able to capture heterogeneity. To do so, a ring-inflation experimental set-up was developed in which a transverse slice of an artery was loaded in the radial direction, while the displacement was estimated from images recorded by a high-speed video camera. The performance of the set-up was evaluated using seven rubber samples and validated with uniaxial tensile tests. For four healthy porcine carotid arteries, material properties were estimated using ultrasound strain imaging in whole-vessel-inflation experiments and compared to the properties estimated with the ring-inflation experiment. A 1D axisymmetric finite element model was used to estimate the material parameters from the measured pressures and diameters, using a neo-Hookean and Holzapfel–Gasser–Ogden material model for the rubber and porcine samples, respectively. Reproducible results were obtained with the ring-inflation experiment for both rubber and porcine samples. Similar mean stiffness values were found in the ring-inflation and tensile tests for the rubber samples as 202 kPa and 206 kPa, respectively. Comparable results were obtained in vessel-inflation experiments using ultrasound and the proposed ring-inflation experiment. This inflation set-up is suitable for the assessment of material properties of healthy vascular tissue in vitro. It could also be used as part of a method for the assessment of heterogeneous material properties, such as in atherosclerotic plaques.

**Electronic supplementary material:**

The online version of this article (10.1007/s10237-020-01292-w) contains supplementary material, which is available to authorized users.

## Introduction

Rupture of atherosclerotic plaques in the carotid artery is a major (15–20% of all cases) cause for stroke (Chaturvedi et al. 2005). Surgical removal of carotid plaques, endarterectomy, is the standard treatment for patients with severe carotid atherosclerosis. Currently, the severity of the stenosis is used to determine whether this procedure is necessary. A plaque resulting in a cross-sectional lumen area reduction larger than 70% and less than 100% is treated. However, lumen narrowing may not be the most important parameter to estimate the risk of plaque rupture. Severely stenosed arteries might actually have a stable plaque, resulting in an overtreatment of patients (Rothwell and Warlow 1999). Moreover, some vulnerable plaques rupture without having a significantly narrowed lumen (Falk et al. 1995). In fact, other parameters, such as plaque geometry, cap thickness and mechanical properties of the plaque components, play an important role in plaque vulnerability. Therefore, the use of biomechanical models to assess rupture risk has become increasingly popular (Cardoso et al. 2014; Chai et al. 2013; Heiland et al. 2013; Holzapfel and Sommer 2004; Lawlor et al. 2011; Speelman et al. 2011). The material properties of the plaque components strongly influence the outcome of these models, and as a result, it is of great importance to describe them accurately (Akyildiz et al. 2011; Finet et al. 2004).

Several methods to determine material properties of human atherosclerotic plaques have been proposed. Uniaxial tensile (Holzapfel and Sommer 2004; Lawlor et al. 2011; Loree et al. 1994; Maher et al. 2009) and planar shear (Mulvihill et al. 2013) tests have been used to characterize plaque tissue. Other groups have also studied this, see, for example, the review of Walsh et al. (2014) and references therein. The samples were cut open, classified based on composition and stretched in the circumferential direction until rupture. However, physiological loading is caused by the blood pressure in the radial direction, and given the fact that plaque tissue is highly anisotropic, the tissue behaviour might be different in the radial direction. Moreover, only global stiffness values were obtained in these previous studies, whereas the properties of the different plaque components are desired in order to make an accurate model.

In compression tests by Lee et al. (1991, 1992) and Maher et al. (2009, 2011), the material was loaded in the radial direction to determine its stiffness. A major limitation of these studies was that the plaque was treated as an isotropic material; therefore, separate determination of the plaque constituents was not feasible.

Another technique, which enables material property assessment on a local level, is micro-indentation. A small spherical indentor is used to apply a force on the tissue, while measuring the indentation depth. The stiffness values of the different plaque components can be determined in the axial direction (Chai et al. 2013). However, for indentation in the radial direction, the tissue needs to be classified into different regions (e.g. fibrous tissue, partially calcified fibrous tissue and calcification) and again global properties of the sample are obtained, instead of the individual component stiffness parameters (Barrett et al. 2009; Ebenstein et al. 2009).

Whole-vessel-inflation tests using ultrasound have been performed to assess plaque properties ex-vivo at physiological loading conditions. Strain maps can be obtained through intravascular ultrasound elastography, allowing for a clear distinction between the hard and soft plaque constituents (Baldewsing et al. 2004; De Korte et al. 2000). However, the large difference in acoustic impedance between soft tissue and calcifications may result in acoustic shadowing. To overcome this problem, Boekhoven et al. (2014) developed a method in which the plaque tissue could be rotated, while an ultrasound probe, placed over the sample, recorded tissue displacement during pressurization at each rotation angle. A drawback of ultrasound-based methods is the difficulty to distinguish between different soft tissue components of the plaque.

**Fig. 1:**
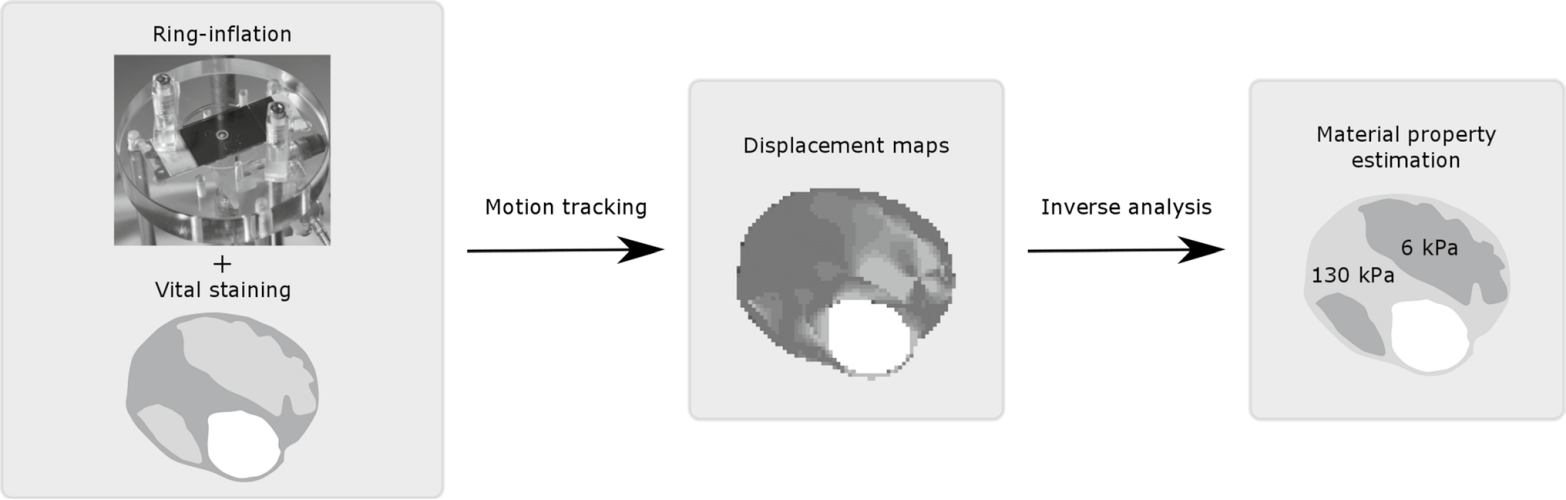
Schematic overview of the methodology. This study is focussed on the ring-inflation experimental part. The images of the plaques were kindly provided by Dr. F. Gijsen from the Erasmus MC, Rotterdam, the Netherlands

Beattie et al. (1998) used a latex tube to inflate 5-mm-thick atherosclerotic rings from the luminal side. A CCD camera system was used to track silicon carbide particles, distributed on the sample, during pressurization. The different plaque components were identified through histology; however, a method in which these components are directly visualized might be preferred. All of these above-mentioned techniques have their strengths and limitations. None of them load the specimen in a physiological way and preserve sample integrity while enabling detailed assessment of the deformation field. Our goal is to develop an experimental set-up to assess material properties of vascular tissue, while applying physiological loading and being able to capture heterogeneity. Therefore, a ring-inflation experimental set-up is proposed in which a slice of an artery is modelled as a 2D object and loaded in the radial direction (Fig. 1). Vital stainings can be applied on the sample to highlight different plaque constituents. A high-speed video camera records the displacements of the sample during pressurization after which deformation fields can be computed by motion tracking. The material properties of the plaque constituents are subsequently assessed from the displacement fields through inverse analysis as done by, for example, Meuwissen (1998).

Due to differences in boundary conditions between such an experimental set-up and the in vivo situation, the main focus of this study is to assess and validate the performance of the set-up itself. Therefore, experiments are performed on rubber and fresh carotid arteries, instead of atherosclerotic tissues. For these less complex materials, the material properties are determined using the pressure–diameter relation, instead of deformation fields obtained by the use of vital stainings and motion tracking. The results from the rubber samples are validated using uniaxial tensile tests. The results of the experiments on healthy arterial tissue are compared to whole-vessel-inflation tests on the same arteries using ultrasound strain imaging.

## Materials and methods

### Ring-inflation experiment

Porcine material was obtained from the slaughterhouse, in accordance with the EC regulations 1774/2002 for the use of slaughterhouse material for research, supervised by the Dutch government (Ministry of Agriculture, Nature and Food Quality) and approved by the Netherlands Food and Consumer Product Safety Authority (NVWA). Common carotid arteries from presumably healthy pigs of 100 kg in weight and either sex were prepared and stored at $-\,20\,^\circ \mathrm{C}$ for later use.

**Fig. 2:**
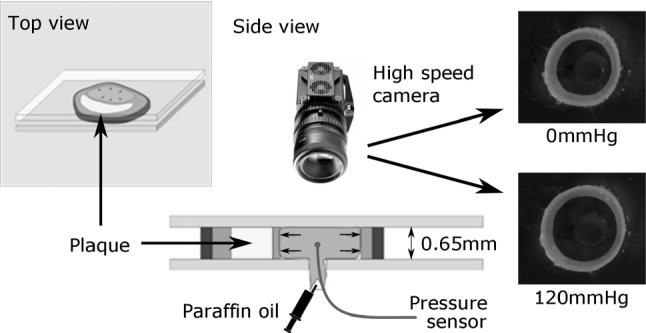
Schematic overview of the experimental set-up

The frozen arteries were cut into 0.7-mm slices using a vibratome (VT1000S, Leica Microsystems GmbH, Nußloch, Germany). The slices were placed in a phosphate-buffered saline solution, stored at $-\,20\,^\circ \mathrm{C}$ and slowly thawed at room temperature at which the experiments were performed as well. Subsequently, the samples were mounted horizontally between two parallel microscope slides and kept 0.65 mm apart through calibrated stainless steel strips, thus yielding an axial compression of the samples of 7% (Fig. 2).

**Fig. 3:**
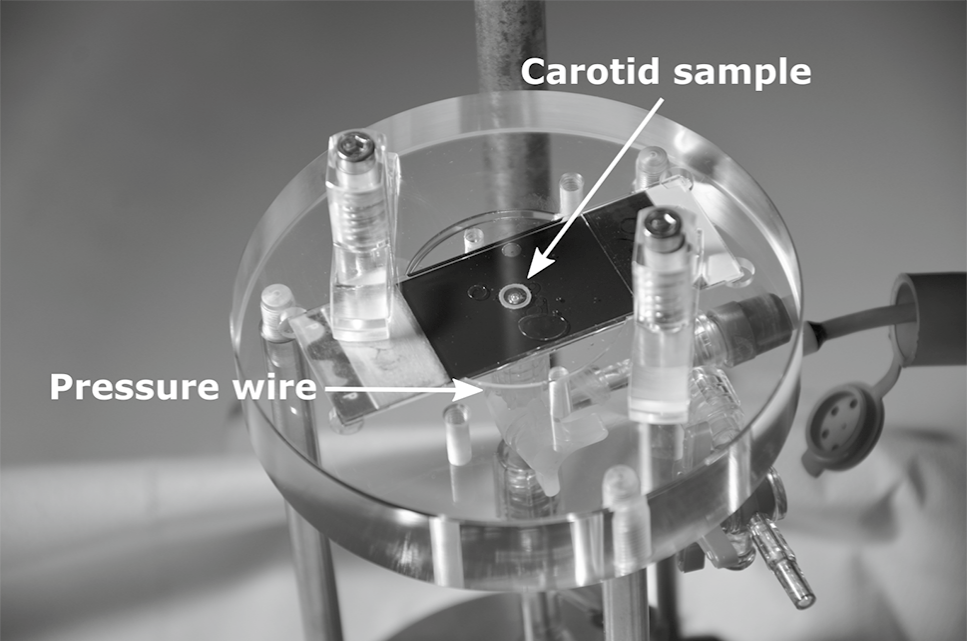
Photograph of the ring-inflation set-up. The tip of the pressure wire is positioned in the lumen of the carotid sample

By injecting fluid into the lumen of the sample, through a 2 mm hole in the bottom slide, the sample is inflated. The injection was done with a servo-motor-driven piston pump, capable of injecting fluid from 0 to 5.9 ml/s. By retracting the pump’s piston, the sample can be deflated again. As a fluid, transparent paraffin oil was used (18512, Sigma-Aldrich, Zwijndrecht, the Netherlands), which pressurized the sample and lubricated the surfaces sliding over the glass platens at the same time. The action of the piston pump was synchronized with data acquisition and video recordings through LabVIEW software (National Instruments, Austin, TX, USA). The luminal pressure was measured using a pressure wire (St Jude Medical, Uppsala, Sweden), with the distal steering tip cut-off, enabling positioning of the sensor of the wire inside the sample, through the hole in the bottom plate (Fig. 3). The motion of the sample was recorded using a high-speed video camera (M5, IDT Vision, Lommel, Belgium), synchronized with the pressure measurement, acquiring video frames at a rate of 200 frames/s. The resulting images had 512 $\times$ 512 pixels with an approximate field of view of 1 $\times$ 1 cm.

To obtain the diameter as a function of pressure, first, the pictures were converted to black and white images. Next, the edges of the ring were detected, after which the lumen was selected. The diameter was calculated from the lumen area, by assuming the ring to have a circular shape. All processing was done using MATLAB software (MATLAB for 64-bit Windows, R2015a, MathWorks, Natick, MA, USA).

### Friction

To obtain reliable measurements, the friction between the sample and the glass plates needs to be negligible. Therefore, possible occurrence of friction was carefully examined. Considerable dynamic friction might occur when the sample slides against the glass, whereas static friction might require the lumen pressure to reach an initial value before the sample starts inflating.

#### Dynamic friction

To assess the role of dynamic friction between the glass plates and the sample, the experiment was modelled in the finite element package MSC.Marc (2013.1.0, 64-bit Windows, MSC Software, Santa Ana, CA, USA). A 2D axisymmetric part of a ring was modelled using a neo-Hookean material model with a Young’s modulus of 1MPa. An arbitrarily oscillating pressure, between 0 and 170 mmHg, was applied manually on the luminal side of the sample. The bottom side of the sample was in contact with a glass plate, where friction was applied. The Coulomb friction model was used with four different coefficients of friction (COFs): 0.01, 0.005, 0.001 and 0 (no friction).

**Fig. 4:**
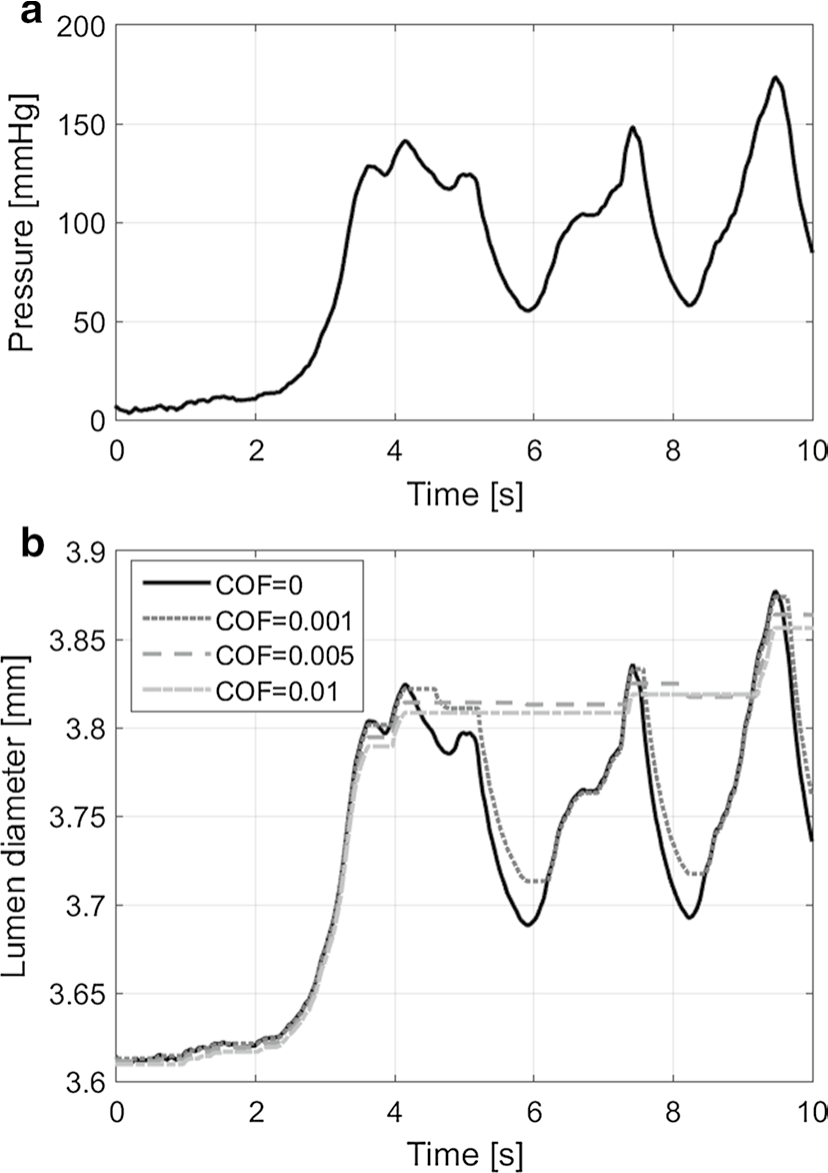
**a** Pressure input for the model. **b** Model output showing the lumen diameter over time using four different coefficients of friction (COFs)

For a COF $\ge 0.005$, the sample still adheres to its surroundings when the pressure decreases (Fig. 4). In our experiment, the sample’s diameter follows the pressure signal in both increasing and decreasing pressures (video data not presented), indicating a negligible COF $\le 0.001$.

#### Static friction

The effect of static friction, caused by compression of the sample, was assessed experimentally. A ring was cut from a sheet of silicone rubber (Wacker Elastosil M 4601 A/B, Wacker Chemie AG, Munich, Germany) and mounted between the glass platens in the experimental set-up. The compression of the sample was varied, by the use of steel strips with different thicknesses between the glass plates, resulting in an axial compression of 0%, 2%, 4%, 7% and 9%. Lumen pressure was increased until a maximum was reached (i.e. leakage of the fluid between the sample and glass platens preventing further pressure increase), after which the pressure was released and subsequently returned to 0 mmHg.

**Fig. 5:**
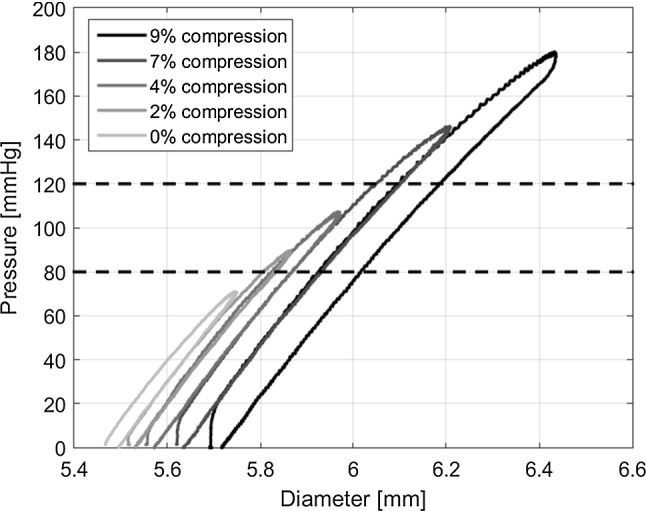
Pressure–diameter curves obtained from the ring-inflation experiment, using five different axial compression levels. The horizontal black dashed lines depict the normal physiological blood pressure of 80–120 mmHg

A higher degree of compression resulted in a higher maximum pressure in the lumen, at the expense of an increasing level of static friction (Fig. 5). To be able to reach physiological pressures (80–120 $\mathrm{mmHg}$) as well as minimizing the static friction, a compression of 7% was chosen.

### Phantom validation

For validation purposes, small rings ($n = 7$) were excised from the same sheet of silicone rubber. The rings, with an inner radius ($r_\mathrm{i}$) of $5.50\,\pm \,0.07\,\mathrm{mm}$, wall thickness of $1.01\,\pm \,0.05\,\mathrm{mm}$ and a thickness of $0.77\,\pm \,0.04\,\mathrm{mm}$, were compressed ($7\%$) between the glass plates and pressurized from 0 to $77\,\pm \,23\,\mathrm{mmHg}$. A solution of 20% soap (regular dishwashing detergent) in water was used instead of paraffin oil, for better lubrication between the rubber sample and the glass plates. The material properties of the rubber were determined with an inverse estimation method. A 1D axisymmetric thick-walled finite element model was used based on Van der Horst et al. (2012). In this parameter estimation procedure, the difference between the pressure–diameter signal from the experiment and the model is minimized. The axial pre-stretch, the unloaded cross-sectional area and the pressure, all obtained from the experiments, were used as input for the model. The material model parameters and the inner radius in the unloaded configuration were optimized using a subroutine in MATLAB (‘fmincon’). Residual stresses were not taken into account. For these rubber samples, an incompressible neo-Hookean material model (Eq. 3c) was used to fit the data, with an initial parameter set of {$c_1 = 200\,\mathrm{kPa}$, $r_i = 5.4\,\mathrm{mm}$}, upper bounds of {300 kPa, 5.8 mm} and lower bounds of {100 kPa, 5.0 mm}.

Furthermore, tensile tests (Zwick/Roell Z010, Ulm, Germany) were performed on rectangular samples ($n = 12$) of the same material. These samples, having a length of $81.2\,\pm \,0.1\,\mathrm{mm}$ and a width of $17.1\,\pm \,0.4\,\mathrm{mm}$, were subjected to 10% strain at a rate of 1% s$^{-1}$, while the extension force was measured using a 20 N load cell. Assuming incompressibility, the true stress ($\sigma _\mathrm{true}$) was calculated:

1 $$\begin{aligned} \sigma _\mathrm{true} = \frac{F}{wd}\lambda \end{aligned}$$

with *F* the applied force (in N), *w* the original sample width, *d* the original sample thickness (in m) and $\lambda$ the stretch. Finally, the stiffness ($c_1$) was obtained from the best fit of the uniaxial, incompressible, neo-Hookean material model to the experimental data, using the following strain energy density function ($\psi$):

2 $$\begin{aligned} \psi = \frac{1}{2}c_1(I_1-3) \end{aligned}$$

with $I_1$ the first invariant of the right Cauchy–Green deformation tensor. The stiffness values found in the inflation tests and the tensile tests were compared using an unpaired two-sample *t*-test with significance level $\alpha = 0.05$.

### Validation on fresh carotid samples

**Fig. 6:**
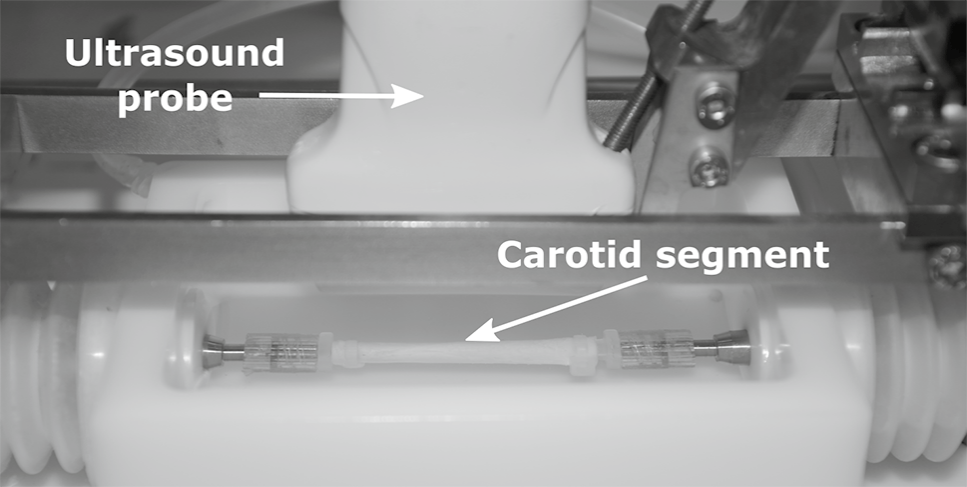
Photograph of the vessel-inflation set-up. The ultrasound probe recorded the displacements of the carotid sample during inflation by the pump

To validate the ring-inflation method using fresh porcine carotid arteries, ultrasound measurements in a mock circulation set-up, based on Van den Broek et al. (2011), were taken on the arteries ($n = 4$) prior to cutting them into slices for the ring-inflation experiment. From each artery, a segment of 20–30 $\mathrm{mm}$ in unstretched length was cut and mounted in a water bath at room temperature (Fig. 6), and stretched to 60% in the axial direction to mimic the in vivo situation (Boekhoven et al. 2014). A servo-operated pump was used to pressurize the sample. A MyLab70 ultrasound scanner (Esaote Europe, Maastricht, the Netherlands) was used for image acquisition. A linear array (LA523, $f_c = 4{-}13$ MHz) ultrasound probe was placed over the sample to measure the position of the arterial walls, while the pressure was measured by a pressure sensor (P10EZ, Becton, Dickinson, Franklin Lakes, NJ, USA). The pressure in the sample was increased linearly from 0 to 120 mmHg in 4–5 s, while the probe recorded 38 lines/cm raw RF data at a frame rate of 73 frames/s in 2D B-mode. The measurement was repeated three times for each artery, without unmounting the sample, to assess reproducibility. The carotid lumen diameter during pressurization was obtained from the RF data using a motion estimation technique by Lopata et al. (2009). It may be noted that a relatively low pressurization speed was used that is not comparable to the in vivo situation. However, the aim of this study was to validate the ring experiment, rather than mimicking in vivo loading conditions.

To obtain samples for the ring-inflation experiment, a slice of 0.7 mm thickness was cut from each artery. Next, the samples were mounted in the experimental set-up at a slight axial compression of 7%. The pressure in the lumen was increased at a constant rate, up to at least 120 mmHg, while the camera captured tissue displacement at a frame rate of 200 fps. For reproducibility reasons, each sample was inflated three times. The samples were repositioned in the set-up after every measurement.

The rings of vessel were cut perpendicular to the vessel axis. A healthy artery is a layered orthotropic structure having a number of fibrous tissue layers in the radial direction, while in axial and circumferential direction these layers extend along the principal coordinate axes. Furthermore, the size of the sample is sufficiently small, so we can assume the shape of the sample to be cylindrical and the material to be homogeneous in axial and circumferential direction. Compression and subsequent inflation of the sample therefore induce a 3D deformation field which is homogeneous in axial direction, while in radial and circumferential direction the deformation state depends on the radial coordinate only. Also, the load case represents typical physiological loads on a piece of vascular tissue in situ (axial deformation combined with internal pressurization). This warrants the description of the 3D deformation field using a 1D model along the radial axis, while simultaneously rendering physiologically relevant data.

Using the previously described inverse parameter estimation model (Sect. 2.3), the pressure–diameter curves from both the vessel- and the ring-inflation experiments were fitted with a Holzapfel–Gasser–Ogden (HGO) model (Holzapfel et al. 2000). The strain energy density function ($\psi$) of this well-known model describes a cross-ply of parallel anisotropic collagen fibre sheets ($\psi _\mathrm{coll}$) embedded in an isotropic matrix ($\psi _\mathrm{mat}$):

3a $$\begin{aligned} \displaystyle \psi&= \psi _\mathrm{mat}+\psi _\mathrm{coll} \end{aligned}$$

3b $$\begin{aligned} \displaystyle \psi _\mathrm{mat}&= \frac{1}{2}c_1(I_1-3) \end{aligned}$$

3c $$\begin{aligned} \displaystyle \psi _\mathrm{coll}&= \frac{k_1}{2k_2}\sum _{i=4,6}^{.}\{\exp [k_2(I_i-1)^2]-1\} \end{aligned}$$

with $c_1$ representing the matrix stiffness (in kPa), $k_1$ the collagen fibre stiffness (in kPa), $k_2$ the nonlinear stiffening of the collagen fibres at higher pressures (dimensionless), and $I_4$ and $I_6$ the orientations of the collagen fibres (in radians). An initial parameter set of {$c_1 = 60\,\mathrm{kPa}$, $k_1 = 4\,\mathrm{kPa}$, $k_2 = 20$, $r_i = 1.4\,\mathrm{mm}$} was used with upper bounds of {$c_1 = 500\,\mathrm{kPa}$, $k_1 = 100\,\mathrm{kPa}$, $k_2 = 200$, $r_i = 1.8\,\mathrm{mm}$} and lower bounds of {$c_1 = 5\,\mathrm{kPa}$, $k_1 = 0.1\,\mathrm{kPa}$, $k_2 = 0.1$, $r_i = 1.0\,\mathrm{mm}$}. The fibre angle, $\beta$, was set to $36^{\circ }$ in the model at physiological pressures from 80 to 120 mmHg, which was found to be fixed for this type of tissue, by Van der Horst et al. (2012). Given the difference in boundary conditions between the vessel- (60% axial stretch) and ring-inflation ($7\%$ axial compression) experiments, the fibre angle in the undeformed state, resulting from the model of the ultrasound experiment, $\beta _0$ (between $22^{\circ }$ and 24$^{\circ }$), was used as input for the ring-inflation model.

## Results

### Phantom validation

**Fig. 7:**
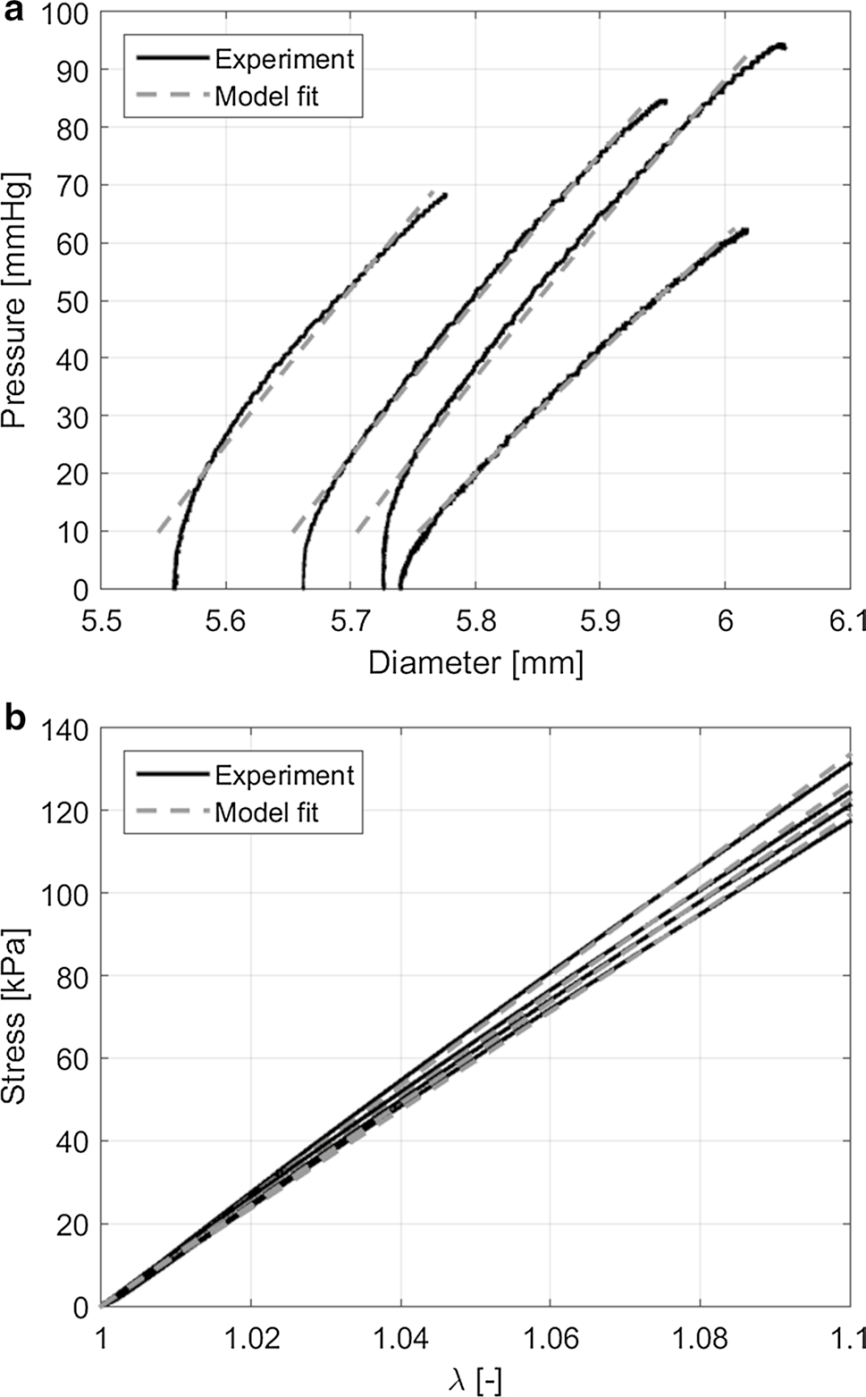
**a** Pressure–diameter curves from the ring-inflation experiment (solid lines) and corresponding model results (dashed lines) for four rubber samples. **b** Stress–stretch curves from the tensile test experiment (solid lines) and model results (dashed lines) for four rubber samples

Pressure–diameter curves from the ring-inflation and tensile test of four randomly selected rubber samples are shown in Fig. 7. All samples show the characteristic strain-softening behaviour for rubber at higher pressures. For six out of seven samples used for the ring-inflation experiment, the diameter did not increase when increasing the pressure from 0 to 10 mmHg. Due to this expected static friction, the experimental data at pressures $> 10\,\mathrm{mmHg}$ were used as input for the parameter estimation model. All twelve samples used for the tensile tests have similar stress–stretch behaviour. Therefore, for the sake of clarity, only four results are shown in Fig. 7.

**Fig. 8:**
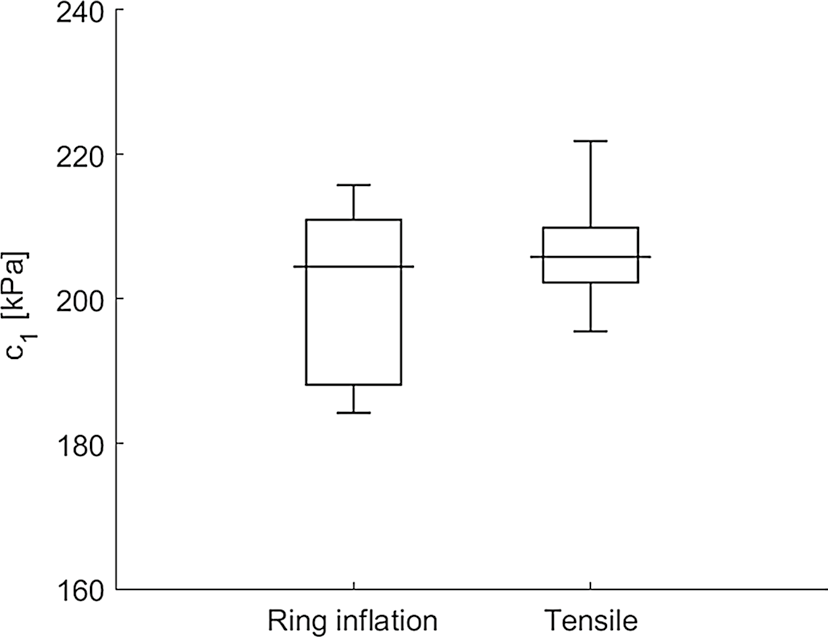
Box and whisker plot of the stiffness ($c_1$) estimation of the rubber in the inflation and tensile tests

Similar stiffness values are found for the rubber samples using the two methods (Fig. 8). A mean $c_1$ of 202 kPa and 206 kPa is obtained for the inflation and tensile tests, respectively. The unpaired two-sample *t*-test confirms the methods result in the same stiffness, $p = 0.28 > 0.05$. A larger spread in stiffness appears to be found in the inflation experiment compared to the tensile test; however, the difference in extreme values (i.e. whisker-to-whisker range) for ring-inflation (31 kPa) and tensile test (26 kPa) is similar.

### Validation on fresh carotid samples

**Fig. 9:**
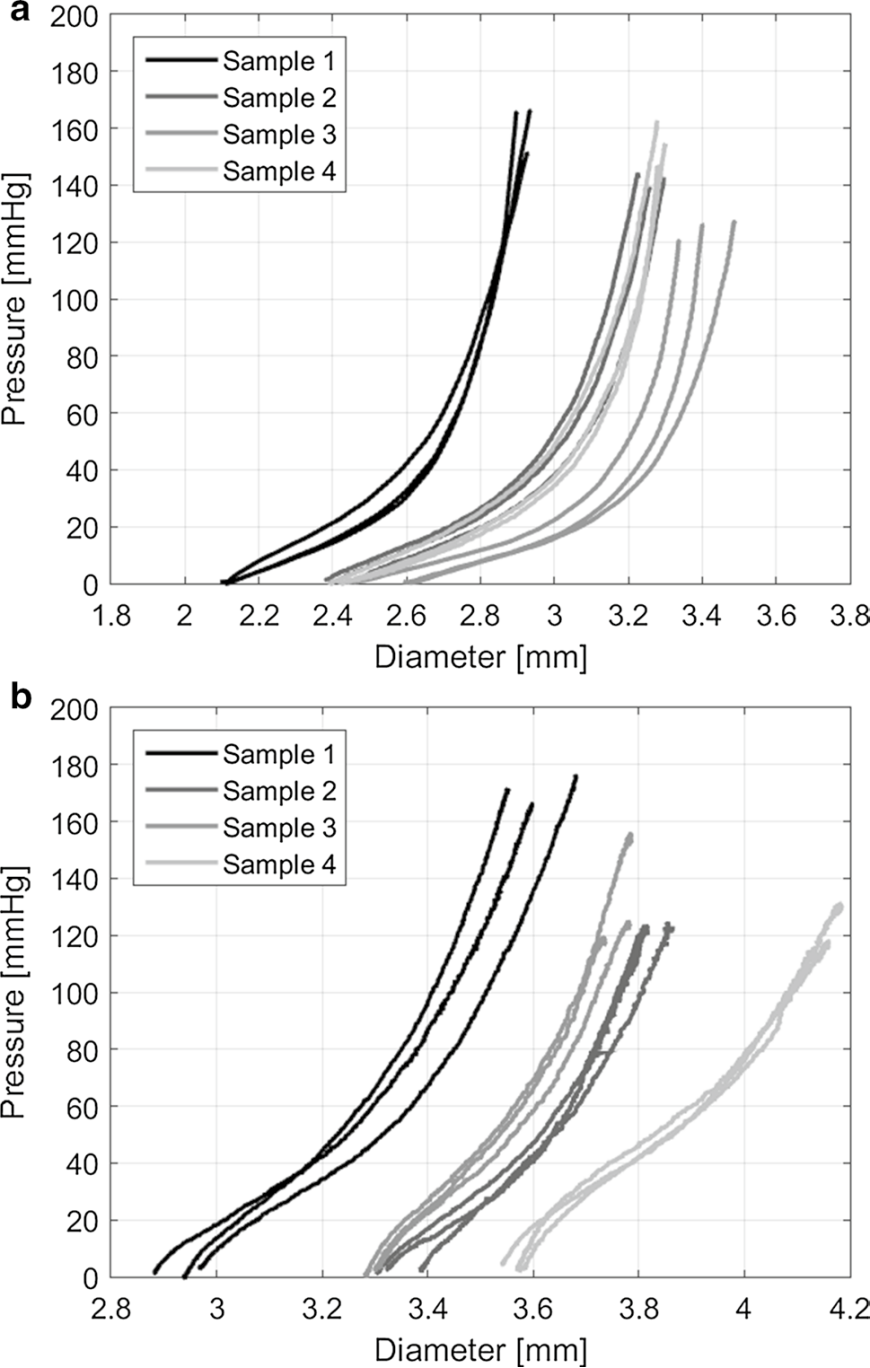
Pressure–diameter curves for the three repeated measurements of the four different arteries. **a** Raw data vessel-inflation experiment. **b** Raw data ring-inflation experiment

Pressure–diameter curves from the vessel-inflation experiment are shown in Fig. 9a. The curves of the four different samples have a similar shape. Moreover, only small differences are found when repeating the experiment with the same sample ($< 258\,\upmu \mathrm{m} \ \sim$ one wavelength of the acquired ultrasound data).

Reproducible results were obtained in the ring-inflation experiment (Fig. 9b). Unlike with rubber phantoms, static friction between the glass plates and the sample appears to be negligible for the carotid samples, as the diameter increases immediately after pressurization. However, the shape of the pressure–diameter curve in the ring-inflation experiment, where the sample is under a slight axial compression, is different compared to the vessel-inflation experiment, in which the sample is axially stretched. The material appears stiffer at low pressures (0–30 mmHg), compared to higher pressures (30–100 mmHg). A video of the inflation of a carotid sample is included as supplementary file.

**Fig. 10:**
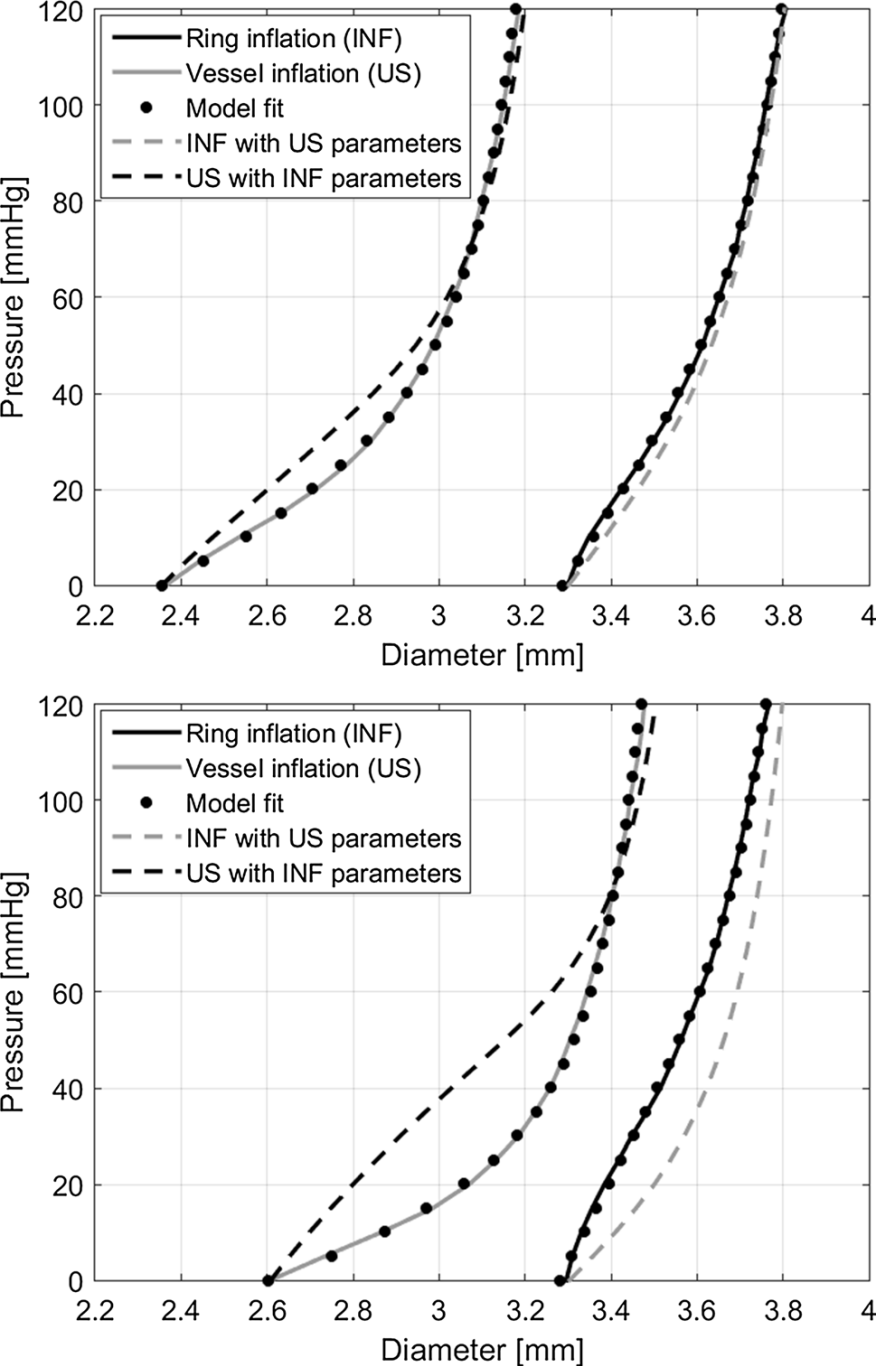
Comparison between the vessel- and ring-inflation experiments for two samples. The model (black dots) is fitted on the raw vessel-inflation (black solid line) and raw ring-inflation data (grey solid line). The grey dashed line shows the model result using the vessel-inflation parameters with the ring-inflation boundary conditions. The black dashed line shows the model result using the ring-inflation parameters with the vessel-inflation boundary conditions

The HGO model can describe the curves from both experiments properly (Fig. 10); however, the parameters found in both experiments do not agree. In general, a higher $c_1$ and lower $k_1$ are found in the ring-inflation test compared to the vessel-inflation test (Table 1). No clear difference is found in $k_2$. As the matrix stiffness ($c_1$) and fibre stiffness ($k_1$) are dependent on each other, the model was rerun, interchanging the parameters between experiments. In other words, the ring-inflation parameters were used as input with the boundary conditions of the vessel-inflation experiment and vice versa. The resulting curves differ in shape; however, the curves do overlap and reveal the same diameter range.

**Table 1:** Material parameter estimates of the Holzapfel–Gasser–Ogden material model for both the vessel- and ring-inflation experiments. The estimates are depicted as mean ± the standard deviation

	Vessel-inflation			Ring-inflation		
Sample	$c_1$ (kPa)	$k_1$ (kPa)	$k_2$ (–)	$c_1$ (kPa)	$k_1$ (kPa)	$k_2$ (–)
1	18.4 ± 5.7	12.1 ± 0.9	19.1 ± 0.6	31.3 ± 5.9	5.0 ± 1.7	12.2 ± 2.3
2	23.2 ± 0.9	12.5 ± 0.8	20.9 ± 2.2	33.0 ± 14.3	12.8 ± 5.5	19.8 ± 5.5
3	17.5 ± 3.0	10.4 ± 2.5	26.6 ± 6.1	65.4 ± 4.8	3.5 ± 0.7	31.6 ± 2.1
4	19.7 ± 2.5	14.4 ± 1.1	19.5 ± 1.8	70.3 ± 10.6	1.2 ± 1.2	29.1 ± 10.6

## Discussion

In this paper, a novel experimental set-up, for the characterization of mechanical properties of vascular tissue, is introduced and validated. This set-up allows the sample to be pressurized in the physiological direction in a quasi-2D setting. The performance of this set-up was successfully verified using rubber phantoms and healthy porcine carotid arteries.

Similarities exist between this ring-inflation method and previously discussed inflation methods; however, there are some notable differences. The ring-inflation experiments performed by Beattie et al. (1998) required the mapping of histology onto the tracking results, which is prone to errors for highly heterogeneous samples. In this method, heterogeneities would directly be visualized on the images. The ultrasound-based inflation methods, as used by Boekhoven et al. (2014), lack the ability to distinguish between different soft tissue components of plaques, whereas in this method, the use of vital staining techniques could overcome this problem.

An important part in the experimental set-up is the role of friction between the sample and the glass plates. The presence of friction would complicate the assessment of the material properties of the tissue. Therefore, the choice of injection fluid is of great importance, as it not only pressurizes the sample, but also serves as a lubricant. During pressurization, the height of the sample decreases, causing the injection fluid to flow between the glass plates and the sample, lubricating the surface, thereby minimizing the dynamic friction. Our numerical model indeed showed that, for both the phantom and the porcine tissue, there is a negligible dynamic friction (COF $\le 0.001$) between the glass plates and the sample, as the sample’s diameter follows the pressure signal at both increasing and decreasing pressures. Paraffin oil lubricated the surface well as no static friction was observed during the porcine carotid inflation experiment. However, paraffin oil was not a suitable lubricant for the rubber–glass interface in the phantom inflation experiment. The soap water solution was a better lubricant for the rubber; however, it was not able to completely prevent stick slip (static friction). The required lubrication film was formed easier in the carotid samples than in the rubber specimens. Nevertheless, similar stiffness values were found for the rubber samples, using the ring-inflation and tensile tests, validating our method. An initial pressure of about 10 mmHg was needed before the sample started to inflate. Past that point, typical neo-Hookean pressure–diameter curves were obtained when inflating the rubber rings. Therefore, it was chosen not to include the data up to 10 mmHg for the estimations of the material parameters. The resulting stiffness estimates were comparable to those estimated from the tensile test, confirming the ring-inflation set-up is suitable for the estimation of material properties.

Samples were assumed to be perfectly circular in shape. This is a limitation of the study, since, especially at low pressure ($<\,30\,\mathrm{mmHg}$) at the start of the inflation test, the shape changes from slightly irregular to circular. In order to minimize this effect, prior to the measurement, the ring was inflated to at least 100 mmHg and slowly deflated to 0 mmHg. However, for repeated measurements on the same sample, the estimated starting diameters varied between 15 and 80 $\upmu \mathrm{m}$ (3–15% of the change in diameter during inflation) at a resolution of $\pm \ 25\,\upmu \mathrm{m}$, influencing the shape of the curve and the estimated material parameters. Apart from this initial inflation and slow deflation, no further preconditioning was performed prior to the measurement. When comparing the pressure–diameter curves from repeated measurements, no possible effect of preconditioning was observed within the accuracy of our method, thus dismissing the need for it.

The estimated material parameters obtained in this study are comparable to the parameters found by Auricchio et al. (2014). In that study, several material models, such as the HGO model, were used to fit experimental data from the works of Delfino (1996) and Sommer et al. (2010). In both works, intact specimens of common carotid artery were axially pre-stretched and loaded by an internal pressure. Delfino used three fixed axial stretches of 1.05, 1.10 and 1.15 with an internal pressure ranging from 0 to 180 mmHg. In Sommer’s work, the specimens had an initial pre-stretch of 1.0–1.3 (in increments of 0.05). The applied axial force was held constant during the internal loading of the vessel from 0 to about 250 kPa. The estimated material parameters were $c_1 = 34.2\,\mathrm{kPa}$, $k_1 = 10.9\,\mathrm{kPa}$, $k_2 = 28.3$ and $c_1 = 26.5\,\mathrm{kPa}$, $k_1 = 20.9\,\mathrm{kPa}$, $k_2 = 56.5$ for the experimental data of Delfino and Sommer, respectively. In this study, the average material parameters for the ring-inflation experiment ($c_1 = 50\,\mathrm{kPa}$, $k_1 = 6\,\mathrm{kPa}$, $k_2 = 23$) are in the same range. Overall, however, a higher matrix stiffness and lower fibre stiffness are found in this study. This could be due to the fact that, besides the dependency of the parameters, in this study, lower pressures (0–120 mmHg) were used for the material parameter estimations. The matrix stiffness plays a larger role in the overall material stiffness at lower pressures, pushing the parameter estimations towards more matrix-dominated material behaviour, rather than material behaviour expressing higher fibre contributions.

The ring- and vessel-inflation measurements on porcine carotid arteries were found to be reproducible and within the same pressure–diameter range. Schulze-Bauer et al. (2003) performed inflation experiments on human iliac arteries under different axial pre-stretches (0–16%) and were able to describe the resulting curves with a single set of parameters. It was therefore expected that a slight axial pre-compression of 7% could still be described with the same set of parameters as in the pre-stretched state. However, in this study, the shape of the pressure–diameter curves from the ring- and vessel-inflation experiments differs, especially in the range of 0–30 mmHg at which a strain-softening behaviour is observed in the ring-inflation tests. Similar shaped pressure–diameter curves were obtained by Delfino (1996) during the inflation of human common carotid arteries. This difference in material behaviour at low pressures resulted in different parameter sets for the ring- and whole-vessel-inflation methods. By interchanging the parameter sets of the ring-inflation and vessel-inflation experiment, similar curves and end point stiffness values were obtained. In other words, similar material properties were obtained, despite the difference in boundary conditions between the experiments (i.e. 7% axial compression and 60% axial stretch). The ring-inflation experiment therefore correctly assesses material properties of healthy carotid arteries. In future work, heterogeneous properties, like in atherosclerotic plaque material, may be assessed. Vital staining techniques could be used to distinguish and characterize different tissue components without affecting their mechanical behaviour. Motion tracking can be performed on these images to obtain deformation maps of the sample, from which the material properties of the plaque constituents can be assessed.

## Conclusion

In this study, a ring-inflation method was proposed for mechanical characterization of vascular tissue. Similar stiffness values were obtained using silicone rubber phantoms in the inflation set-up compared to tensile tests. Moreover, comparable results were obtained in vessel-inflation experiments using ultrasound and the proposed ring-inflation experiment. This inflation set-up is suitable for the assessment of material properties of healthy vascular tissue. It could also be used as part of a method for the assessment of heterogeneous material properties, such as in atherosclerotic plaques.

## Electronic supplementary material

Below is the link to the electronic supplementary material.

Supplementary material 1 (avi 1470 KB)
